# Supplementary figures and images for: Immune Cells in the Spleen of Mice Mediate the Inflammatory Response Induced by Mannheimia haemolytica A2 Serotype
Source: Animals (Basel). 2024 Jan 19;14(2):317. doi: 10.3390/ani14020317 (PMC10812571; doi:10.3390/ani14020317)

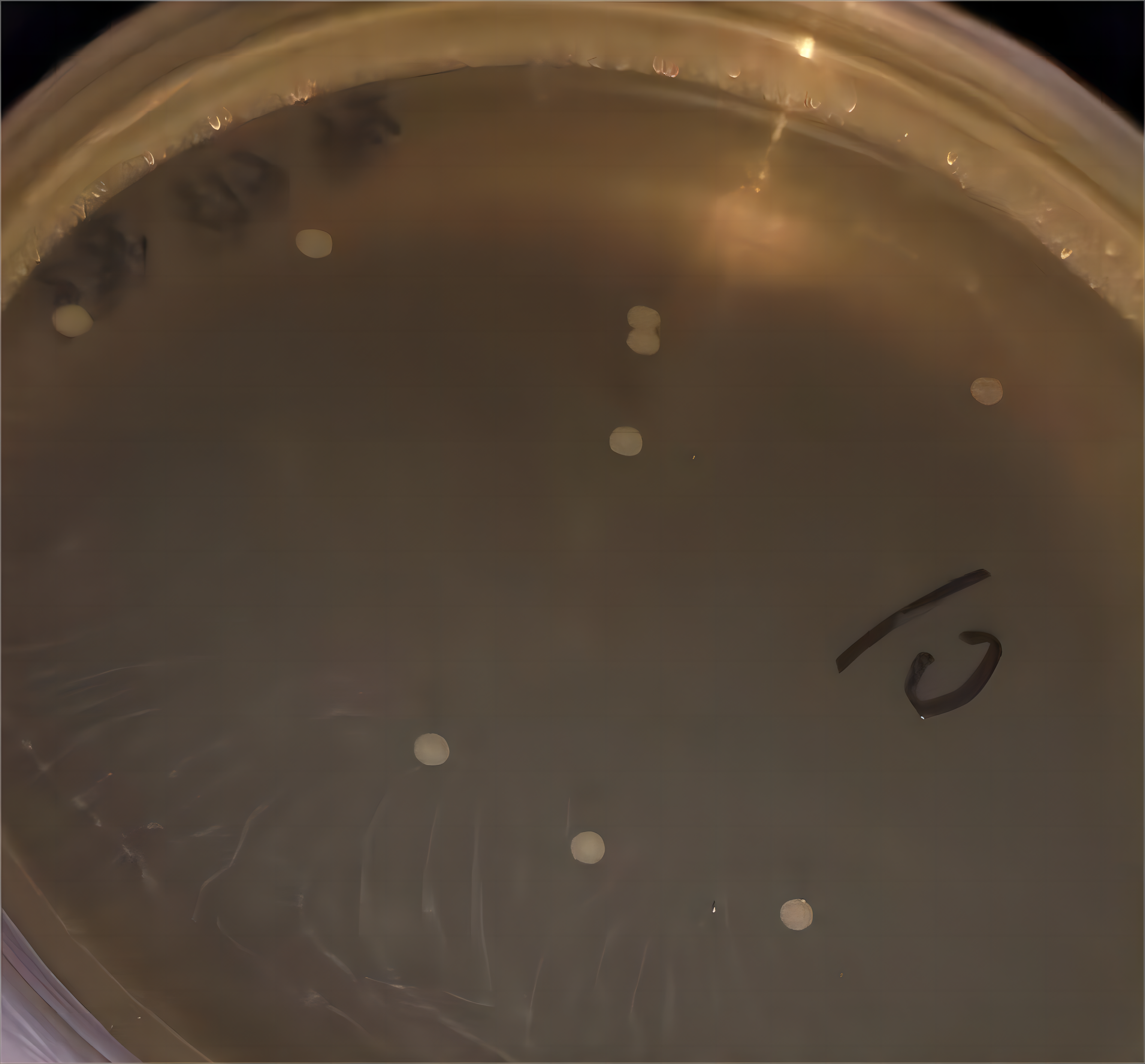

Supplement: Supplementary file 1 [file animals-14-00317-s001.zip › Figure S1.png]
